# Incorporation of Hydroxyeicosatetraenoic Acid Isomers into Macrophage Phospholipids Reveals Class-Specific Distribution

**DOI:** 10.3390/biom16050692

**Published:** 2026-05-07

**Authors:** Alvaro Garrido, Patricia Monge, Natalia Pérez, María A. Balboa, Jesús Balsinde

**Affiliations:** 1 Bioactive Lipids and Lipidomics Core, IBGM, CSIC-UVA, 47003 Valladolid, Spain; 2 Centro de Investigación Biomédica en Red de Diabetes y Enfermedades Metabólicas Asociadas (CIBERDEM), Instituto de Salud Carlos III, 28029 Madrid, Spain; 3 Lipid Metabolism and Inflammation Group, IBGM, CSIC-UVA, 47003 Valladolid, Spain

**Keywords:** hydroxyeicosatetraenoic acid, phospholipid fatty acid incorporation, inflammation, macrophages

## Abstract

Phospholipid fatty acid incorporation and remodeling are central processes through which immune cells adapt their membranes during activation. Macrophages are known to integrate oxidized fatty acids into phospholipids, yet the principles governing this distribution remain incompletely defined. Hydroxyeicosatetraenoic acids (HETEs) are abundant products generated during inflammation, and their integration into membrane phospholipids may influence signaling, trafficking, and membrane organization. Although individual HETE isomers differ in biosynthesis and function, it is not known whether macrophages handle them differently. Here, we address how 5-, 12-, and 15-HETE are incorporated into murine peritoneal macrophage phospholipids during inflammatory stimulation. We show that each isomer exhibits a distinctive phospholipid-class distribution, with 12-HETE preferentially entering choline phospholipids (PC), 15-HETE enriching phosphatidylinositol (PI), and 5-HETE distributing more broadly across PC, PI and ethanolamine phospholipids (PE). All three isomers are incorporated predominantly at the sn-2 position and showed similar molecular species distribution within each class, with diacyl PC, PE plasmalogens, and PI(18:0/HETE) serving as dominant acceptors. RAW264.7 cells reproduce these patterns. In ether phospholipid-deficient RAW.108 cells, incorporation into ether species is lost but compensated by increased routing into diacyl PC and PE, while PI incorporation remains unchanged. Collectively, these findings reveal that phospholipid class, not simple availability, determines where HETEs are incorporated. This distribution is preserved across macrophage cell types and remains intact even when ether phospholipids are absent, indicating that class specific pathways, rather than lipid subclass composition, primarily determine HETE incorporation.

## 1. Introduction

Macrophages are key regulators of inflammation and tissue remodeling through their ability to generate a wide array of oxidized lipid mediators derived from polyunsaturated fatty acids [1,2,3,4]. Among these, arachidonic acid (AA) is especially abundant in macrophage phospholipids and is rapidly mobilized upon cellular activation. Once released, macrophages convert a substantial fraction of the fatty acid into diverse eicosanoids, including prostaglandins, leukotrienes, and various hydroxyeicosatetraenoic acids (HETEs) [1,2,3,4,5,6,7,8,9,10,11,12,13,14,15,16,17,18,19,20,21,22,23]. Importantly, a considerable portion of the liberated AA is efficiently reincorporated back into phospholipids, underscoring the importance of reacylation pathways within the Lands cycle in controlling the amount of free fatty acid available for eicosanoid synthesis [24,25,26,27].

In addition to free unmetabolized AA, macrophages also avidly incorporate HETEs into phospholipids [28,29,30,31,32,33]. This results in the formation of a phospholipid pool containing an oxidized fatty acyl chain. Such incorporation may influence multiple biophysical properties of the membrane, thereby modulating cellular processes. Emerging evidence further suggests that these oxidized phospholipids may also exert biological activities on their own [34,35,36,37]. Moreover, this esterified pool of HETEs could also be mobilized at later stages of the activation process, potentially providing a reservoir for delayed or sustained signaling.

HETEs are generated by several lipoxygenases acting on free AA, and their relative abundance depends not only of the nature of the stimulus applied to the macrophages, but also on their anatomical localization. In mice, peritoneal macrophages are the only cells that express substantial amounts of the two major enzymes responsible for HETE synthesis, namely 5-lipoxygenase and 12/15-lipoxygenase [38,39]. Despite their considerable structural homology, these two lipoxygenases generate distinct products and are independently regulated rather than functionally coupled. 5-Lipoxygenase produces 5-*S*-hydroxyeicosatetraeonic acid (5-HETE), while 12/15-lipoxygenase produces both 12-*S*-hydroxyeicostetraenoic acid and 15-*S*-hydroxyeicostetraenoic acid (15-HETE) [38,39,40,41,42,43,44,45,46,47,48].

The biological roles of 5-, 12-, and 15-HETE are diverse and context-dependent. 5-HETE is a precursor for leukotriene biosynthesis and functions as a chemoattractant and pro-inflammatory mediator, enhancing leukocyte recruitment and supporting classical macrophage activation. 12-HETE influences cytoskeletal dynamics, migration, and angiogenesis, contributing to macrophage behavior in wound healing and tumor microenvironments. In contrast, 15-HETE is associated with anti-inflammatory and pro-resolving pathways; it participates in the biosynthesis of specialized pro-resolving mediators such as lipoxins, and it can modulate transcriptional programs that favor tissue repair. Together, these metabolites constitute a lipid signaling axis that enables macrophages to respond adaptively to changing physiological demands [38,39,40,41,42,43,44,45,46,47].

The esterification of exogenous HETEs into macrophage membrane phospholipids represents a major and largely overlooked gap in oxylipin biology, as HETEs are often assumed to remain unesterified and to act primarily through receptor-mediated or transcriptional mechanisms. As a result, the biological functions of oxidized phospholipids containing hydroxy fatty acids remain poorly understood. Critically, the phospholipid classes and molecular species that preferentially incorporate exogenous HETEs have seldom been examined in a systematic manner, despite its potential implications for membrane remodeling and oxylipin signaling. In this report, we show that macrophages possess a substantial capacity to incorporate HETEs into discrete cellular phospholipids, and this process exhibits striking acceptor specificity. Identifying distinct pathways for HETE incorporation may offer insight into how macrophages coordinate inflammation and its resolution.

## 2. Materials and Methods

### 2.1. Reagents

Cell culture medium was from Corning (Glendale, AZ, USA). Organic solvents (Optima^®^ LC/MS grade) were from Fisher Scientific (Madrid, Spain). Lipid standards were from Cayman (Ann Arbor, MI, USA) or Larodan (Malmö, Sweden). Deuterated 5-*S*-HETE(d_8_), 12-*S*-HETE(d_8_), and 15-*S*-HETE(d_8_) were from Cayman. All other reagents were from Sigma-Aldrich (Madrid, Spain).

### 2.2. Cell Culture

Resident peritoneal macrophages from Swiss male mice (University of Valladolid Animal House, 10–12 week old) were obtained by peritoneal lavage using 5 mL cold phosphate-buffered saline, as described elsewhere [48]. The cells were plated at 2 × 10^6^ per well (6-well plates) in 2 mL RPMI 1640 medium with 10% heat-inactivated fetal bovine serum, 100 U/mL penicillin, and 100 μg/mL streptomycin, and allowed to adhere for 20 h in a humidified atmosphere of 5% CO_2_ at 37 °C. Wells were extensively washed to remove nonadherent cells. Adherent macrophages were then used for experimentation. Cells were placed in serum-free medium for 1 h, then challenged with 0.5 mg/mL yeast-derived zymosan for the indicated times in the absence or presence of deuterated HETEs at 1 µM, added in ethanol. Controls received the same volume of ethanol, which never exceeded 0.1%. Zymosan, a particulate extract of the *Saccharomyces cerevisiae* cell wall, contains β-glucans and mannans that engage pattern-recognition receptors including Dectin-1, TLR2/TLR6, and mannose receptors, thereby triggering robust phagocytic and inflammatory responses in macrophages [49]. In brief, zymosan particles were suspended in phosphate-buffered saline, boiled for 60 min, and washed three times. The final pellet was resuspended in phosphate-buffered saline at 20 mg/mL and stored frozen [50]. For opsonization, the particles were treated with heat-inactivated mouse serum (10 mg zymosan per 1 mL serum) for 20 min at 37 °C. Since heating the serum inactivates complement factors, the opsonized zymosan produced in this manner promotes IgG-mediated responses [51,52,53]. Zymosan aliquots were diluted in serum-free medium and sonicated before addition to the cells. No phospholipase A_2_ activity was detected in the zymosan batches used in this study, as assessed by in vitro activity assay [54,55,56]. A commercial kit based on the Bradford procedure [57] was used to measure cell protein content (BioRad Protein Assay, Bio-Rad, Hercules, CA, USA). All procedures involving animals were carried out under the supervision of the Institutional Committee of Animal Care and Usage of the University of Valladolid (Approval No. 7406000) and are in accordance with the guidelines established by the Spanish Ministry of Agriculture, Food, and Environment and the European Union.

RAW264.7 macrophage-like cells and their ether phospholipid-deficient variant RAW.108 (generously provided by Dr. R. A. Zoeller, Boston University, Boston, MA, USA) [58,59,60], were grown in Dulbecco’s modified Eagle’s medium supplemented with 10% (*v*/*v*) fetal bovine serum, 100 U/mL penicillin, 100 g/mL streptomycin, and 2 mM L-glutamine at 37 °C in a humidified atmosphere of 5% CO_2_, as previously described [61,62].

### 2.3. Liquid Chromatography/Mass Spectrometry (LC-MS) Analyses of Phospholipids

Samples were matched for cell protein before lipid extraction and analysis. The following internal standards were added: 15 pmol each of 1,2-dipalmitoyl-*sn*-glycero-3-phosphoinositol, 1,2-diheptadecanoyl-*sn*-glycero-3-phosphoethanolamine, and 1,2-diheptadecanoyl-*sn*-glycero-3-phosphocholine. Lipids were extracted according to Bligh and Dyer [63].

The lipid fraction was re-dissolved in hexanes/isopropanol (30:40, *v*/*v*) and injected into a Thermo Scientific Dionex Ultimate 3000 high-performance liquid chromatograph, equipped with an Ultimate HPG-3400SD standard binary pump and an Ultimate ACC-3000 autosampler column compartment (Waltham, MA, USA). Separation was carried using a FORTIS HILIC (150 × 3 mm, 3 µm particle size) (Fortis Technologies, Neston, UK). The mobile phase consisted of a gradient of solvent A (hexanes/isopropanol 30:40, *v*/*v*) and solvent B (hexanes/isopropanol/20 mM ammonium acetate in water, 30:40:7, *v*/*v*/*v*). The gradient started at 75% A from 0 to 5 min, then decreased from 75% A to 40% A at 15 min, from 40% A to 5% A at 20 min, holding at 5% until 40 min, increasing to 75% at 41 min. Then the column was re-equilibrated holding 75% A for an additional 14 min period before the next sample injection [64]. The flow rate through the column was fixed at 0.4 mL/min. The liquid chromatography system was coupled online to an AB Sciex QTRAP 4500 mass spectrometer equipped with a Turbo V ion source and a TurbolonSpray probe for electrospray ionization (AB Sciex, Framingham, MA, USA). Source parameters were set as follows: ion spray voltage, −4500 V; curtain gas, 30 psi; nebulizer gas, 50 psi; desolvation gas, 60 psi; temperature, 425 °C. Phospholipid species were analyzed in scheduled multiple reaction monitoring mode (MRM) with negative ionization, detecting in Q3 the *m*/*z* 327.2, corresponding to HETE with 8 deuterium atoms, as [M-H]^−^. Compound parameters were fixed as follows: declustering potential; −45 V (choline glycerophospholipids), −60 V (ethanolamine glycerophospholipids) −30 V (phosphatidylinositol), −50 V (phosphatidylserine), −60 V (phosphatidic acid), −50 V (phosphatidylglycerol); collision energy: −50 V (choline glycerophospholipids), −40 V (ethanolamine glycerophospholipids), −60 V (phosphatidylinositol), −50 V (phosphatidylserine), −45 V (phosphatidic acid), −45 V (phosphatidylglycerol); entrance potential, −10 V; and collision cell exit potential, −8 V. All glycerophospholipids were detected as [M-H]^−^ ions except choline glycerophospholipids, which were detected as [M+CH_3_COO]^−^ ions. Quantification was carried out by integrating the chromatographic peaks of each species and comparing with the peak area of the internal standards that corresponded to each class [65,66]. MRM transitions for the internal standards were as follows: PC(17:0/17:0), 820.6 → 269.2; PE(17:0/17:0), 718.5 → 269.2; PI(16:0/16:0), 809.5 → 255.2.

### 2.4. Analysis of Positional Specificity

Cell extracts containing deuterated HETEs esterified into phospholipids were incubated with bee venom phospholipase A_2_ (Sigma-Aldrich) to assess positional specificity [67]. The phospholipid substrates were dispersed as sonicated vesicles in a reaction buffer consisting of 50 mM Tris-HCl (pH 8.0) and 5 mM CaCl_2_, and incubations were carried out for 90 min at 37 °C. Reactions were quenched by addition of organic solvent, and the HETE-containing phospholipid content in the samples was analyzed by LC–MS as described above.

### 2.5. Statistical Analysis

The results are shown as means ± standard error of the mean. In experiments using peritoneal macrophages, each data point represents an independent preparation generated by pooling cells collected from multiple mice. Statistical significance was analyzed by *t*-test (two groups) or by ANOVA (more than two groups), followed Tukey’s post hoc test, using SigmaPlot software, version 14.0 (Systat Software Inc., San Jose, CA, USA). A value of *p* < 0.05 was considered statistically significant.

## 3. Results

**Scheme 1:**
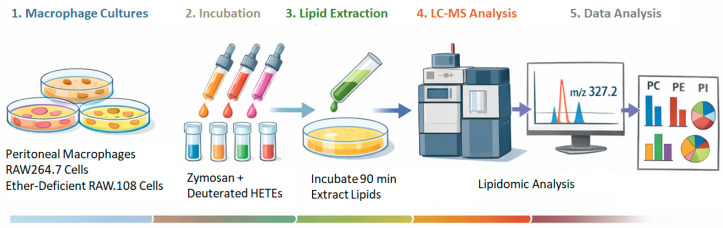
Experimental setup for HETE incorporation studies. Workflow illustrating macrophage preparation, zymosan stimulation and addition of deuterated HETEs, incubation, lipid extraction, LC–MS analysis, and quantification of incorporated HETE into phospholipids.

Cells are known to take up HETEs from exogenous sources and incorporate them into phospholipids [28,29,30,31,32,33]. However, no comparative studies have been carried out to examine the incorporation of the different isomers or the specific molecular species into which they incorporate. To address this gap, we designed the present study in which parallel cultures of murine peritoneal macrophages were exposed to equal amounts of deuterated 5-HETE, 12-HETE, and 15-HETE at the time they were stimulated with yeast-derived zymosan. This stimulus has long been used as a model for investigating pathways involving phospholipase A_2_-mediated phospholipid fatty acid turnover in macrophages [5,6,7,8,9,10,11,12,13,14]. Because the zymosan used in this study is IgG-opsonized, macrophage activation proceeds primarily through Fcγ receptors, which signal via Syk and downstream protein kinase C and mitogen-activated protein kinase pathways that converge on cytosolic phospholipase A_2_α activation and robust AA release [68,69]. Thus, the use of zymosan is particularly suitable for our study, as it increases the pool of lysophospholipid acceptors available for fatty acid incorporation [21,70,71,72]. Scheme 1 provides a graphical description of the experimental setup used to stimulate macrophages and assess HETE incorporation.

**Figure 1:**
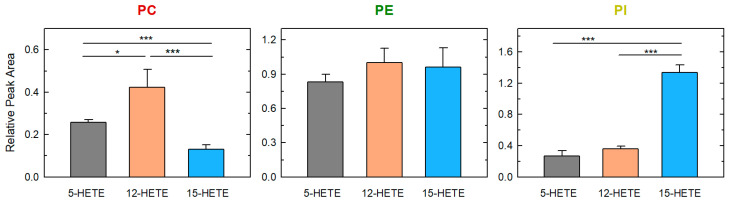
Incorporation of HETEs into macrophage phospholipid classes. Peritoneal macrophages were stimulated with 0.5 mg/mL zymosan in the presence of 1 µM deuterated 5-HETE, 12-HETE, or 15-HETE for 90 min, as indicated. Afterward, the incorporation of each fatty acid into PC, PE, and PI was determined by LC-MS. Data are shown as means ± standard error of four independent determinations. * *p* < 0.05, and *** *p* < 0.001, significant difference between the indicated treatments.

Figure 1 shows that incorporation of 5-HETE, 12-HETE, and 15-HETE into murine peritoneal macrophage phospholipids occurred in broadly comparable amounts, although differences were observed with regard to the phospholipid classes targeted by each isomer. There was a preference of 12-HETE over 5-HETE and especially over 15-HETE for incorporation into PC. In contrast, 15-HETE showed a strong preference for incorporation into PI compared with 5-HETE and 12-HETE, which tended to compensate for its lower incorporation into PC. Finally, no significant differences were observed among the isomers with respect to incorporation into PE.

**Figure 2:**
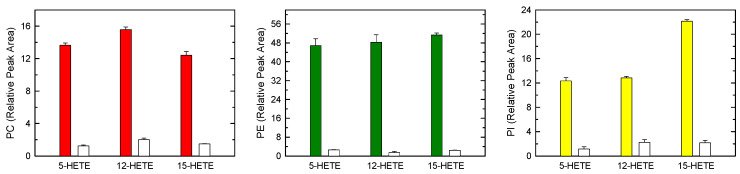
Hydrolysis of HETE-containing phospholipids by bee venom phospholipase A_2_. Cells were stimulated with 0.5 mg/mL zymosan in the presence of 1 µM deuterated 5-HETE, 12-HETE, or 15-HETE, as indicated. Afterward, cellular lipid extracts were prepared and incubated either without (colored bars) or with (open) bee venom phospholipase A_2_. HETE-containing phospholipids were subsequently quantified by LC-MS. Data are shown as means ± standard error of three independent determinations.

To determine whether the different HETE isomers incorporated into phospholipids were esterified at the sn-2 position of the glycerol backbone, lipid extracts from cells incubated with all three deuterated HETE isomers were subjected to hydrolysis by bee venom phospholipase A_2_. As shown in Figure 2, treatment with the enzyme caused a pronounced loss of deuterium-labeled HETE across all major phospholipid classes, with more than 90% of the incorporated label being removed. This extensive loss of label after phospholipase A_2_ digestion is fully consistent with the exogenously supplied HETE isomers being incorporated predominantly at the sn-2 position.

**Figure 3:**
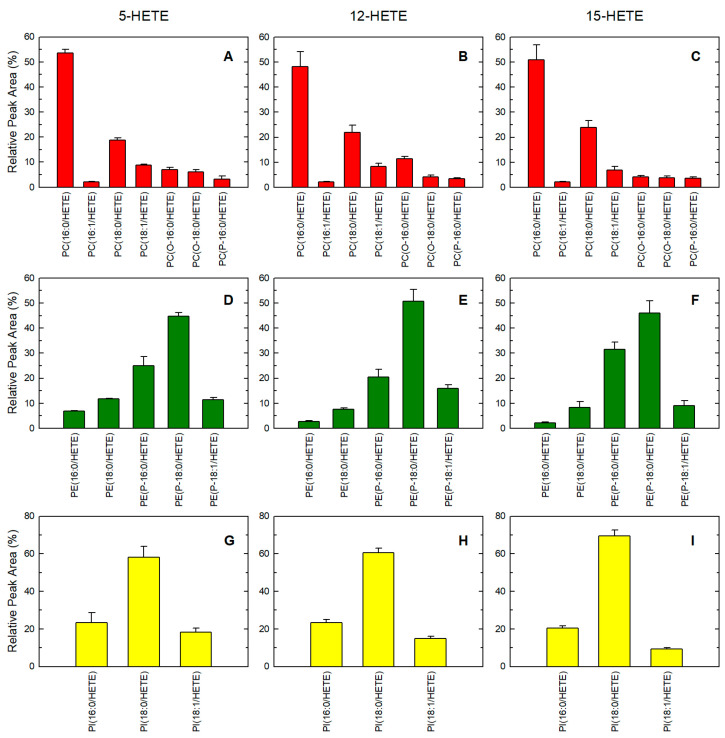
Incorporation of HETEs into phospholipid molecular species of peritoneal macrophages. Cells were stimulated with 0.5 mg/mL zymosan in the presence of 1 µM deuterated 5-HETE (left column), 12-HETE (middle column), or 15-HETE (right column) for 90 min, as indicated. Afterward, the incorporation of each fatty acid into PC molecular species (panels **A**–**C**; red bars) PE molecular species (panels **D**–**F**; green bars), and PI molecular species (panels **G**–**I**; yellow bars) was determined by LC-MS. Data are shown as means ± standard error of four independent determinations.

The distribution of 5-HETE, 12-HETE, and 15-HETE among phospholipid molecular species is shown in Figure 3. According to nomenclature recommendations [73,74,75], the prefix O- before the first fatty chain indicates an ether linkage at the sn-1 position, whereas the prefix P- denotes a plasmalogen with a vinyl ether linkage at sn-1. Fatty acyl chains are abbreviated by the number of carbon atoms followed by the number of double bonds.

The incorporation patterns of the three isomers among molecular species within each phospholipid class were remarkably similar. For PC molecular species, there was a clear preference of all three isomers for incorporation into diacyl species (PC(16:0/HETE) and PC(18:0/HETE)) over those containing an sn-1 ether bond (PC(O-16:0/HETE) and PC(O-18:0/HETE)). This behavior was not observed for the PE molecular species, where the plasmalogen species (PE(P-16:0/HETE) and PE(P-18:0/HETE)) were the preferred targets for incorporation of all three HETE isomers. In the PI class, a single species, PI(18:0/HETE), accounted for the majority of the incorporation.

RAW264.7 cells are widely used as macrophage surrogates because they reproduce key macrophage functions in response to stimuli such as bacterial lipopolysaccharide and yeast-derived zymosan, including phagocytosis and robust lipid turnover and inflammatory signaling [76,77,78,79,80,81]. However, the phospholipid composition of primary macrophages and RAW264.7 differs in several respects, including the level and distribution of AA and other polyunsaturates among certain phospholipid classes [82,83,84]. It was therefore important to determine whether this cell line also constitutes a reliable model for studies on stimulated phospholipid fatty acid incorporation and remodeling.

**Figure 4:**
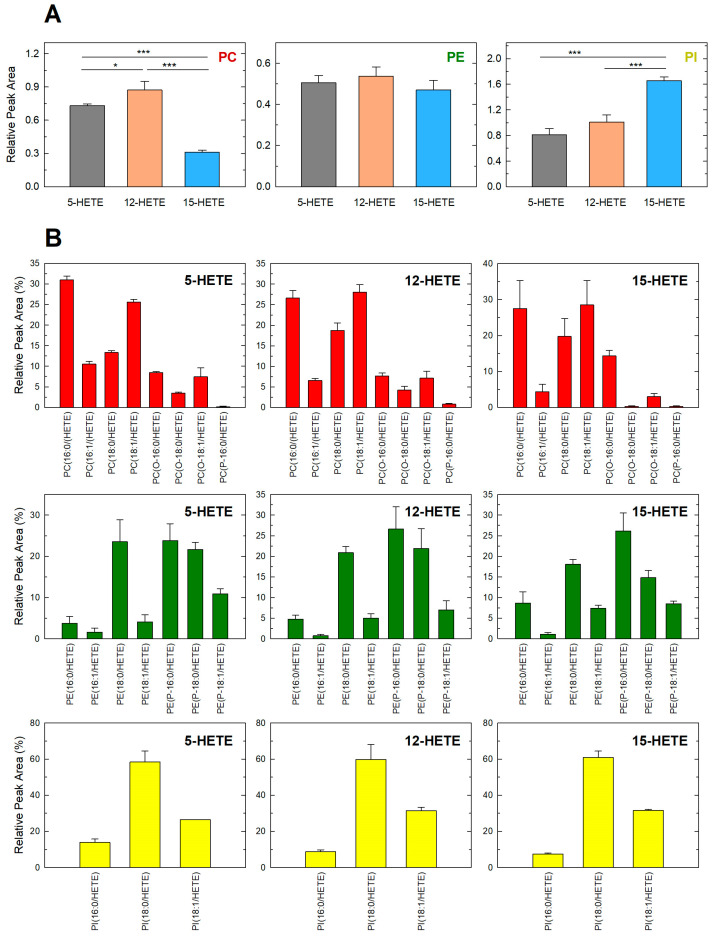
Incorporation of HETEs into RAW264.7 macrophage-like cell phospholipids. Cells were stimulated with 0.5 mg/mL zymosan in the presence of 1 µM deuterated 5-HETE, 12-HETE, or 15-HETE for 90 min, as indicated. Afterward, the incorporation of each fatty acid into phospholipid classes (PC, PE and PI) (**A**) or specific molecular species (PC species in red, PE species in green, PI species in yellow) (**B**) was determined by LC-MS. Data are shown as means ± standard error of four independent determinations. * *p* < 0.05, and *** *p* < 0.001, significant difference between the indicated treatments.

Figure 4A shows that RAW264.7 cells exposed to either 5-, 12-, or 15-HETE under identical conditions as those used for primary macrophages, displayed incorporation patterns that were highly concordant with those observed in primary macrophages. Across all three HETE isomers, the hierarchy of phospholipid-class preference was essentially the same. Thus, 12-HETE was incorporated predominantly into PC, 15-HETE showed a strong and selective enrichment in PI, and 5-HETE distributed more broadly across PC, PE, and PI without pronounced differences.

Concordance between RAW264.7 cells and peritoneal macrophages was also evident with respect to molecular species distribution (Figure 4B). The same molecular species of PC, PE, and PI species that accumulated HETEs in primary peritoneal macrophages were likewise the major acceptors in RAW264.7 cells, and the relative proportions followed comparable patterns. These parallels suggest that the enzymatic machinery responsible for acyl chain remodeling operates under similar conditions in both systems. Thus, the determinants of HETE incorporation appear to be intrinsic features of macrophage lipid metabolism rather than peculiarities linked to cell origin or differentiation state. The close correspondence between RAW264.7 cells and primary macrophages therefore validates the use of RAW264.7 cells as a reliable model for dissecting the structural rules that govern the incorporation of oxidized fatty acids into membrane phospholipids. By extension, the data reinforce the suitability of RAW264.7 cells as a relevant in vitro model for macrophage studies.

A striking feature of these results is the finding that PE plasmalogens are major acceptors for HETE incorporation, suggesting a role for this particular kind of phospholipids in determining how HETE isomers are distributed among molecular species. The strong preference for PE plasmalogens, together with the distinct behavior of ether-linked PC species, raises the question of how HETE incorporation proceeds in cells that lack ether phospholipids. It was therefore necessary to examine a model system in which ether-linked species are absent, in order to assess whether the incorporation patterns observed in primary macrophages depend on the presence of plasmalogens or reflect more general features of phospholipid remodeling. For these studies, we used the RAW.108 cell line, a variant of RAW264.7 cells established by Zoeller and co-workers that lacks ether phospholipids due to a deficiency of peroxisomal dihydroxyacetone phosphate activity [58,59,60,85]. We have recently confirmed the near-complete absence of ether phospholipids in these cells by mass spectrometry-based lipidomic analysis [62].

**Figure 5:**
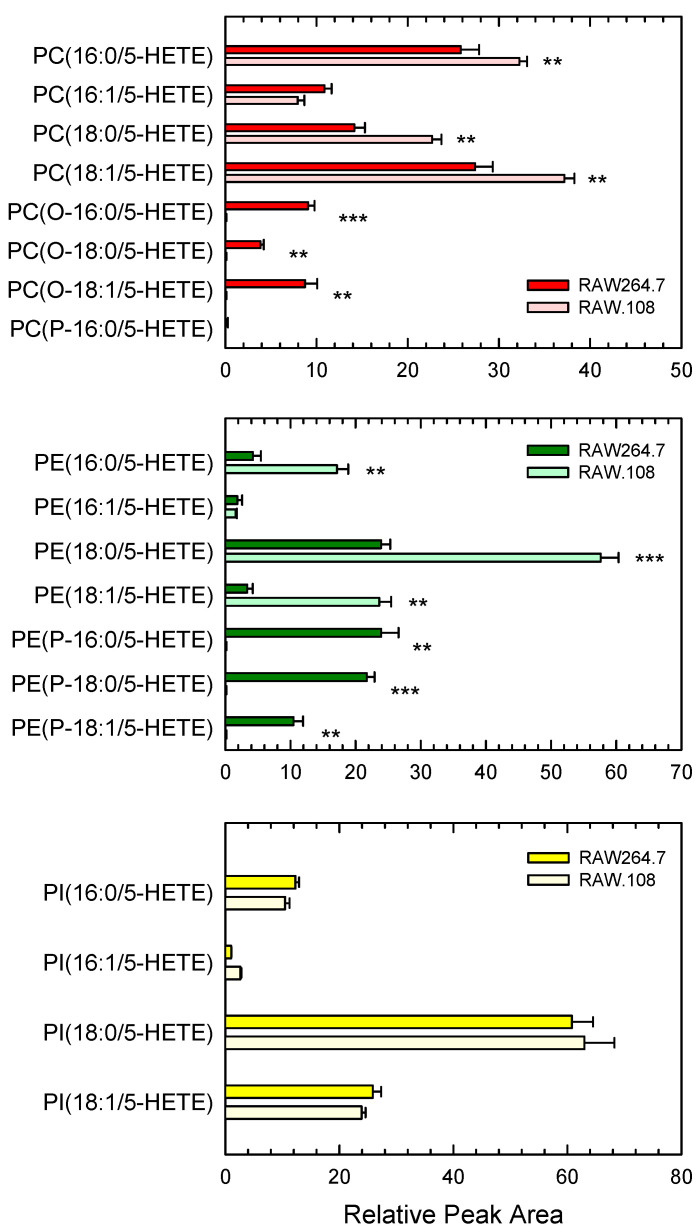
Incorporation of 5-HETEs in plasmalogen-deficient cells. RAW264.7 cells and their ether phospholipid-deficient variant RAW.108 were stimulated with 0.5 mg/mL zymosan in the presence of 1 µM deuterated 5-HETE for 90 min, as indicated in the figure. Afterward, the incorporation of each fatty acid into phospholipid molecular species (PC species in red, PE species in green, PI species in yellow) was determined by LC-MS. Data are shown as means ± standard error of three independent determinations. ** *p* < 0.01, and *** *p* < 0.001, significantly different from the corresponding species in RAW264.7 cells.

Given that the distribution of HETE incorporation among molecular species within each phospholipid class was remarkably similar for all three isomers (Figure 3 and Figure 4B), subsequent experiments focused on 5-HETE as a representative substrate. Figure 5 shows the incorporation of 5-HETE into the phospholipid molecular species of ether phospholipid-deficient RAW.108 cells.

As expected for cells lacking ether phospholipids, incorporation into ether-linked PC and PE species was negligible. Instead, 5-HETE was routed into diacyl PC and diacyl PE. The increased incorporation into the diacyl species of PC and PE effectively compensated for the lack of ether phospholipids, such that the amount of fatty acid in the PC and PE classes was preserved with respect to that of wild type RAW264.7 cells. Incorporation of 5-HETE into the PI class was essentially unchanged relative to wild type cells, with PI(18:0/HETE) again accounting for most of the incorporation within this class (Figure 5). The finding that, despite compositional differences at the molecular species level, the overall pattern of incorporation remained broadly similar at the phospholipid-class level, indicates that the basic features of HETE incorporation and remodeling are preserved even in the absence of ether phospholipids.

## 4. Discussion

The present study provides a detailed view of how structurally distinct HETE isomers are incorporated into macrophage phospholipids and how this process is shaped by both the headgroup composition and the presence of ether-linked species. By using primary peritoneal macrophages under conditions that promote phospholipase A_2_–dependent lipid turnover, we were able to directly compare the incorporation behavior of the three major HETE isomers, namely 5-, 12-, and 15-HETE, at both the class and molecular species levels. A striking finding is that the three HETE isomers are incorporated into macrophage phospholipids in broadly comparable amounts, yet they display clear differences in class-level preference. The enrichment of 12-HETE in PC, the strong incorporation of 15-HETE into PI, and the more even distribution of 5-HETE across classes indicate that headgroup identity exerts a substantial influence on the fate of these oxidized fatty acids. At the same time, the prominent contribution of PE plasmalogens in peritoneal macrophages, and the altered distribution observed in their absence, highlight the modulatory role of ether-linked phospholipids in shaping these patterns. Importantly, phospholipase A_2_ digestion confirmed that all three HETEs are incorporated overwhelmingly at the sn-2 position, indicating that they enter the canonical remodeling pathway rather than being accommodated through alternative mechanisms.

Molecular species analysis provided further insight into the structural determinants of HETE incorporation reactions. Despite their different class level preferences, the three isomers exhibited remarkably similar patterns of incorporation among species within each class. In PC, all three HETEs favored diacyl species over ether linked species as acceptors; in PE, plasmalogens were the dominant acceptors; and in PI, a single species, namely PI(18:0/HETE), accounted for most of the incorporation. These features suggest that the remodeling machinery recognizes shared structural features of the HETE isomers and channels them into a limited set of molecular species that are particularly well suited to accommodate oxidized acyl chains. Such an observation aligns with the idea that oxidized fatty acids are not incorporated randomly into membrane phospholipids but instead follow patterns shaped by both enzymatic selectivity and the structural features of the available phospholipid pools. Acyltransferases involved in phospholipid fatty acid recycling exhibit preferences for specific headgroups and acyl chain combinations, and the behaviors of 12-HETE, 15-HETE, and 5-HETE may respond to such selective activities [86,87,88,89,90].

It is interesting to note that the molecular species distribution of incorporated HETEs closely parallels that of endogenous AA-containing phospholipids, which in macrophages are enriched in PC species containing palmitic or stearic acid at the sn-1 position, PE plasmalogens, and PI species bearing stearic acid at the sn-1 position [21]. Together with the predominant sn-2 esterification demonstrated by phospholipase A_2_ digestion, these findings further support the view that HETEs enter the same fatty acid recycling pathways as AA, and that the class-specific incorporation patterns arise from intrinsic enzymatic selectivity rather than lysophospholipid availability. The predominance of diacyl PC species for 12-HETE incorporation is consistent with the activity of acyltransferases selective for polyunsaturated fatty acids acting on lysoPC, with LPCAT3 representing a prime candidate [71]. The selective accumulation of PI(18:0/HETE) strongly implicates MBOAT7, which preferentially reacylates stearoyl- lysoPI with polyunsaturated fatty acids [91]. As for HETE incorporation into PE plasmalogens, to the best of our knowledge no acyltransferase has been definitively shown to prefer 2-lysoplasmalogen substrates; however, some studies have implicated MBOAT5 as a likely contributor to the introduction of polyunsaturated acyl chains, including AA, into plasmalogen PE species [91]. Together, these candidates provide a mechanistic framework for the phospholipid-class specificity observed in our study.

The headgroup-specific preferences observed here may have important consequences for how macrophages utilize oxidized fatty acids during inflammatory activation. Selective routing of 12-HETE to PC or 15-HETE to PI could influence processes such as membrane curvature, signaling complex assembly, and the availability of substrates for further enzymatic transformation [92,93,94,95,96]. In this regard, incorporation into PI is particularly noteworthy, since PI and its phosphorylated derivatives participate in a wide range of signaling pathways that regulate phagocytosis, vesicle trafficking, and cytoskeletal dynamics [97,98]. The strong preference of 15-HETE for this class is consistent with previous studies [28,29,30,33,99,100,101] and suggests that its incorporation may intersect with hitherto unidentified pathways not typically associated with oxidized fatty acids. Conversely, the broader distribution of 5-HETE may reflect a more general role in membrane remodeling rather than engagement with specific signaling routes. These considerations point to the possibility that the structural diversity of phospholipids not only accommodates exogenous HETEs but may also shape their functional impact within activated macrophages.

The experiments with ether phospholipid-deficient RAW.108 cells provide insight into this specific kind of phospholipid in shaping these incorporation patterns. In addition to their role in modulating a number of key cellular responses such as oxidative homeostasis, lipid-driven signaling, and the fluidity and organization of membrane dynamics [102,103,104,105], ether phospholipids, in particular the plasmalogens fraction, are also known to constitute a major reservoir of polyunsaturated fatty acids in phagocytic cells [21]. Thus, in principle, it would be logical to expect that ether phospholipids play an important role in macrophage HETE incorporation. As expected, the absence of ether lipids eliminated incorporation into ether-linked PC and PE plasmalogen species. However, rather than altering the overall distribution pattern, the system compensates by increasing incorporation into the corresponding diacyl species. This compensation preserves the total amount of HETE recovered in the PC and PE classes, demonstrating that the remodeling network reallocates incoming fatty acids to maintain class level balance even when a major subset of molecular species is unavailable. Incorporation into PI is essentially unchanged, with PI(18:0/HETE) remaining the dominant species, underscoring the stability of the PI fatty acid recycling pathway.

To conclude, we should acknowledge a limitation of our study. Deuterated HETEs were supplied exogenously, and zymosan stimulation is known to generate endogenous HETEs that may partially dilute the labeled pool. Within the time frame of our stimulation conditions (up to 90 min), endogenous HETE production is low [6,7,76] and is therefore unlikely to affect our results or conclusions substantially, but it cannot be considered negligible.

## 5. Conclusions

Our work identifies key molecular determinants of how macrophages incorporate exogenous HETE isomers into their membranes. Headgroup identity emerges as the primary determinant of HETE incorporation. Molecular species composition, including the presence or absence of ether bonds, modulates the distribution within each class but does not alter the overall distribution. This robustness suggests that macrophages possess a flexible yet tightly regulated incorporation system that integrates oxidized fatty acids in a manner that preserves both structural integrity and functional specialization. The ability of the system to maintain class level incorporation patterns even in the absence of ether phospholipids highlights the adaptability of the phospholipid fatty acid incorporation machinery and underscores the central role of headgroup-specific pathways in controlling the fate of oxidized fatty acids.

By defining the structural features that guide HETE incorporation, our results provide a foundation for future studies aimed at understanding how oxidized lipids are integrated into macrophage membranes and how these processes influence inflammatory responses and membrane remodeling.

## Data Availability

The original contributions presented in this study are included in the article. Further inquiries can be directed to the corresponding author(s).

## Abbreviations

The following abbreviations are used in this manuscript:

AA	Arachidonic acid
HETE	Hydroxyeicosatetraenoic acid
LC-MS	Liquid chromatography coupled to mass spectrometry
PC	Choline-containing glycerophospholipids
PE	Ethanolamine-containing glycerophospholipids
PI	Phosphatidylinositol
